# Glutathione Redox Activity—An Adaptative Mechanism in Clear Cell Renal Cell Carcinoma

**DOI:** 10.3390/ijms27083509

**Published:** 2026-04-14

**Authors:** Corina-Daniela Ene, Ilinca Nicolae, Cristina Capusa

**Affiliations:** 1 Department of Nephrology and Internal Medicine, Carol Davila University of Medicine and Pharmacy, 020021 Bucharest, Romania; ccalexandr@yahoo.com; 2 Dr. Carol Davila, Clinical Hospital of Nephrology, 010731 Bucharest, Romania; 3 Victor Babes Clinical Hospital of Tropical and Infectious Diseases, 030303 Bucharest, Romania; drnicolaei@yahoo.ro

**Keywords:** glutathione redox homeostasis, cytoprotection, inflammation, oxidative stress, angiogenesis, apoptosis

## Abstract

Environmental, genetic, immunological and metabolic factors are involved in renal cell carcinoma development. Clear cell renal carcinoma (ccRCC) is the most frequent renal cancer, with a complex metabolic physiopathology. The present study focuses on the characterization of chemical changes in glutathione redox homeostasis induced by oxidative damage and their relevance to ccRCC. We developed a prospective, case–control study that included 92 subjects diagnosed with ccRCC by histopathological exam and 40 healthy subjects. In each subject, we evaluated the chemical changes in glutathione redox homeostasis, antioxidative capacity, nitrosative stress, carbonyl stress, inflammation (IL-12 family members, albumin), angiogenesis factors and apoptosis. Compared to the control, in ccRCC subjects, we detected high levels of oxidative/electrophile stress, of hypoxia, and of inflammatory- and angiogenesis-related factors and low levels of anti-inflammatory-, anti-oxidative- and apoptosis-related factors. In ccRCC, positive correlations between glutathione redox homeostasis members expression and electrophile metabolites levels, respectively, angiogenesis markers and inflammatory parameters detected. Negative relations with anti-inflammatory and antioxidant markers were assessed. Glutathione redox homeostasis was altered in ccRCC, functioning as an active redox mechanism, with an essential role in the development and progression of ccRCC.

## 1. Introduction

Stress is an inevitable part of life. Prolonged exposure to chronic stress can lead to cumulative effects, increasing the risk of health problems [1,2]. Epidemiological studies identified associative relationships between exposure to environmental factors, behavioral factors, metabolic and genetic factors, and urological cancers [3,4,5,6,7]. To prevent oxidative damage, cancer cells have developed antioxidant defense systems, such as nicotinamide adenine dinucleotide in its oxidized and reduced forms of NAD+/NADH, nicotinamide adenine dinucleotide phosphate in its oxidized and reduced forms of NADP+/NADPH, and oxidized glutathione as GSH/GSSG. These redox mechanisms are dynamic networks that regulate multiple biological processes of tumors, including the cell cycle, proliferation, invasion, and metastasis [1]. In oncogenesis, GSH/GSSG is an effective mechanism to counteract xenobiotics and electrophilic metabolites. In the kidneys, GSH exerts cytoprotective effects, but bio stimulatory activities have also been described (exposure to cisplatin, metabolism of halogenated alkenes) [8].

In the field of redox biology, glutathione redox homeostasis metabolism plays a fundamental role. The high level of L-GSH (L-γ glutamyl-L-cysteinyl-glycine) in the kidneys is ensured through two directions: GSH possesses exclusive structural characteristics, and the kidneys have unique functional features to maintain renal GSH homeostasis (synthesis, degradation, transport, and redox turnover) [8]. In the structure of GSH, two unique attributes have been identified: the nucleophilic sulfur and the γ-glutamyl isopeptide bond. The first allows GSH to function as an antioxidant and anti-electrophile. The γ-glutamyl isopeptide bond ensures the stability of the molecule in various locations in the body (systemic circulation, bile, tissues, extra- and intracellular compartments) without being degraded. The kidneys are energy-consuming organs, with intense metabolism, exposed to hypoxia, containing numerous mitochondria in the tubular nephrons, and rich in GSH. In renal cells, three mechanisms serve to maintain GSH homeostasis, namely de novo synthesis, absorption from exogenous sources through the plasma membranes, and glomerular filtration and regeneration of GSH by reducing GSSG. De novo GSH synthesis occurs in the liver and kidneys and it is catalyzed by glutamate cysteine ligase (GCL) and glutathione synthase (GS). GCL is a heterodimer controlled by the status of the heterodimer, GSH levels, post-translational modifications (phosphorylation, glycation), translational factors (electrophiles), erythroid factor 2 (Nrf2), mitogen-activated protein kinase (MAPK), muscle aponeurotic fibrosarcoma (MAF), and IKKβ–NF–κB. GS is a homodimer, regulated only by transcriptional expression [9,10]. The second mechanism for managing renal GSH is represented by the kidney’s ability to extract 80% of circulating GSH. Approximately 3/8 of the efflux of GSH from the renal tubules is rapidly broken down by GGT, *ChaC1* and *ChaC2* genes, and dipeptidase. After glomerular filtration and reabsorption in the proximal tubule, the amino acids are used for GSH resynthesis [9,10]. The high level of GSH in the kidneys is also supported by the reduction in GSSG, a reaction catalyzed by GSH reductase (GR) in the presence of NADPH(H+) derived from the pentose phosphate pathway (PPP), which is overexpressed in ccRCC [9,10]. This redox buffer cycle ensures the removal of electrophiles from cells and regulates redox signaling pathways through their interaction with specific target proteins, which constitute the active metabolic–redox networks. In conclusion, the dynamics of GSH in subcellular compartments and in the tumor microenvironment, as well as the translocation from systemic circulation to tissues, are factors that induce and regulate tumor development [11,12].

Renal cancer (RCC) is an aggressive disease that develops from the proximal renal tubular epithelium, with an increasing incidence and mortality rate globally (400,000 new cases annually, 175,000 deaths per year) [4]. Genetic variants of RCC account for 75–80% of all kidney cancers, with a 5-year survival rate of 65–70% [13]. To date, there are over 15 genes associated with hereditary RCC, with a prevalence of 5–8% of all kidney cancers [14]. The most common histological variants of RCC are clear cell RCC von Hipple-Lindau deficient (ccRCC, 75–80%), papillary RCC type 1 *MET* (15–20%), papillary RCC type 2 *FHa* (10%), *TFE3b* or *TFEBb* (10%), and chromophobe RCC *PTEN* (8%.) Rare forms have also been described, such as collecting duct carcinoma, translocation RCC, medullary renal carcinoma, mucinous tubular carcinoma, spindle cell carcinoma, sarcomatous carcinomas, and rhabdoid carcinomas [13,15,16]. The multiomic profile shows that ccRCC is a complex disease, with histological heterogeneity (clear cytoplasm cells, filled with lipids and glycogen), immune (immune infiltration, PD-L1 expression), genetic (mutations in *VHL*, *PBRM1*, *BAP1*, *SETD2*, *KDMSC*), epigenetic (*SETD8* associated with lipid storage, grade, stage, and prognosis of the disease, elevated levels of 2-hydroxyglutarate), metabolic (expression of long non-coding RNAs, altered metabolism of glucose, lipids, amino acids, AMPK signaling, activation of GSH metabolism, disturbances in oxidative phosphorylation, TCA cycle, pentose phosphate pathway, activation of *HIF1a*, *HIF2a*, *NFkB*, *c-Myc*, inactivation of *VHL*), treatment responses (radiotherapy, chemotherapy, immune checkpoint inhibitors), antiangiogenic therapy), and extracellular matrix remodeling (MMPs, TIMPs, epithelial–mesenchymal transition, angiogenesis) [17,18,19,20,21,22,23].

Based on the data presented earlier, we appreciate that ccRCC is a complex disease, with an incompletely deciphered metabolic substrate, in which cellular stress and active redox metabolic circuits play an essential role. The objective of the current study was to identify changes in the glutathione redox markers by determining the oxidation states of GSH in relation to systemic concentrations of reactive oxygen species (ROS), reactive nitrogen species (RNS), their relevance to the pathology of ccRCC, and their clinical influence.

## 2. Results

### 2.1. Glutathione Redox Homeostasis Activity in ccRCC

The glutathione redox activity markers were evaluated by serum levels of reduced and oxidized gluyhathione and their ratios. We detected an increased activity of reactive sulfur species in ccRCC. In ccRCC, GSH was 1.66 fold higher compared to the control (*p* < 0.05) and GSSG was 1.37 fold higher compared to the control (*p* < 0.05), while GSH/GSSG increased 1.22 fold compared to the control (*p* < 0.05) (Table 1).

### 2.2. Reactive Oxygen Species in ccRCC

Oxidative stress was evaluated in ccRCC by antioxidant capacity. ImAnOx was 1.48 fold lower in the ccRCC group (*p* < 0.05) (Table 2).

### 2.3. Nitrosative Stress in ccRCC

The level of nitrosative stress in ccRCC was evaluated by direct nitrite, total nitrite and nitrate. In the ccRCC group, we detected an increase of 2.3 fold for direct nitrite (*p* < 0.05), of 2.59 fold for total nitrite (*p* < 0.05) and of 2.75 fold for nitrate (*p* < 0.05) compared to the control (Table 3).

### 2.4. Carbonyl Stress in ccRCC

Carbonyl stress in ccRCC was evaluated by serum levels of hydroxynonenal and pentosidine. Compared to the control group, 4-HNE increased 2.31 fold (*p* < 0.05), while pentosidine increased 1.53 fold (*p* < 0.05) in ccRCC group (Table 4).

### 2.5. Inflammation in ccRCC

Inflammation was evaluated in ccRCC by serum levels of IL-12 monomers and heterodimers and albumin. When compared to the control, in the ccRCC group, albumin was 1.10 lower (*p* < 0.05). IL-12p70 increased 2.1 fold (*p* < 0.05), IL-12p40 increased 3.1 fold (*p* < 0.05), and IL-23 increased 2.34 fold (*p* < 0.05), while IL-12p35 decreased 1.45 fold (*p* < 0.05) and IL-35 decreased 1.20 fold (*p* > 0.05) (Table 5).

### 2.6. Angiogenesis in ccRCC

Angiogenesis, evaluated by serum levels of HIF-1a, HIF-1b, their ratio and VEGF, was overexpressed in ccRCC. HIF-1a increased 1.86 fold (*p* < 0.05), HIF-1b increased 1.57 fold (*p* < 0.05), their ratio increased 1.14 fold (*p* < 0.05) and VEGF increased 3.44 fold (*p* < 0.05) compared to the control group (Table 6).

### 2.7. Apoptosis in ccRCC

Apoptosis in ccRCC was evaluated by serum levels of caspase-3. Caspase-3 was 1.84 fold higher in the ccRCC group compared to the control (*p* < 0.05) (Table 7).

### 2.8. GSH-GSSG System and Studied Markers in ccRCC

The relation between the metabolites of the GSH-GSSG cycle and the studied markers in ccRCC are presented in Table 8. We assessed a negative relation between sulfur species and antioxidant capacity in ccRCC, statistically significant for ImAnOx. A positive correlation was detected between nitrogen species and markers of glutathione redox homeostasis, statistically significant for total nitrite–GSH, total nitrite–GSH/GSSG, nitrate–GSH and nitrate–GSSG. The carbonyl species correlated positively, which was statistically significant, with GSH–GSSG system members. The members of the IL-12 family had different behaviors in relation with the metabolites of the GSH-GSSG cycle. IL-12p70 correlated positively, statistically significant with GSH, GSSG and their ratio, IL-23 correlated with GSH, while IL-12p40 had no correlation with GSH, GSSG and their ratio. Meanwhile, IL-12p35 and IL-35 correlated negatively with the metabolites of glutathione redox homeostasis in ccRCC. The markers of angiogenesys correlated positively with GSH, GSSG and their ratio, except VEGF and GSH/GSSG, which had no correlation.

## 3. Discussion

The biological relevance and regulatory functions of free radicals and associated metabolites under physiological and pathological conditions are currently being intensively studied in carcinogenesis. In the present study, we documented the serum levels of ROS (ImAnOx), RNS (direct nitrites, total nitrites, nitrate), RCS (4-HNE, pentosidine), RSS (GSH, GSSG, GSH/GSSG ratio) in ccRCC and non-ccRCC subjects. These reactive biological species were overexpressed under hypoxic and oxidative conditions in patients with ccRCC compared to the control (Table 9). The perturbance of the redox balance in ccRCC may be explained by potentialally exposure of the kidney to high concentrations of oxidants and reactive electrophiles, partly caused by increased aerobic metabolism in renal proximal tubules [8]. Additionally, the kidneys are the second energy-consuming organs, with an accelerated metabolic rate and increased mitochondrial density in the tubular nephrons [10]. The modulation of the cellular stress environment in ccRCC is based on the regulation of GSH metabolism [2].

Endogenous reactive species (ROS, RNS, RCS, RSS) and associated metabolites have interesting actions in carcinogenesis. Firstly, their normal or moderately elevated levels positively affect homeostasis by regulating the redox-dependent signaling of cellular processes. In excess, these levels are associated with the exacerbation of oxidative damage and inflammatory responses and alteration of angiogenic and apoptotic mechanisms, thus contributing to the development and progression of ccRCC (Table 9). Although there are remarkable similarities between these endogenous reactive species, RSS is differentiated by their ability to mitigate pathological processes. These effects could be attributed to the metabolic flexibility of RSS, which can be stored, recycled, and are chemically versatile [24,25,26].

GSH, GSSG, and the GSH/GSSG ratio are widely used indicators of cellular redox status and glutathione metabolism. Although these parameters reflect the functional state of the glutathione system, they do not directly measure the activity of the γ-glutamyl cycle, which involves several enzymes and intermediates [27,28]. The GSH-GSSG redox system was profoundly disturbed in renal cancer. Elevated serum levels of GSH, GSSG, and the GSH/GSSG ratio were associated with the levels of toxic/electrophilic/carcinogenic compounds in the body (direct nitrites, total nitrites, nitrates, 4-HNE, pentosidine), with overexpression of inflammation (IL-12 cytokine family), hypoxia (HIF1a, HIF2a), angiogenesis (VEGF), and the limitation of apoptosis (caspase-3) in ccRCC (Table 9).

In these reactions, GSH was oxidized by electrophilic substances and was converted to GSSG via GR. However, the compensatory increase in GSH in renal proximal tubular cells was associated with carcinogenesis [8]. The alteration of extracellular degradation mechanisms of GSH (GGT hydrolyzes GSH to Cys-Gly and glutamate) and intracellular (intracellular glutamylcyclotransferase ChaC1 and ChaC2 specific directly splits GSH inside the cell into Cys-Gly and 5-oxoproline) had a crucial impact on the development and spread of malignant conditions [25,26,27]. Decreased expression of ChaC1 was a poor prognosis indicator of ccRCC (KIRC). ChaC2 acted as a tumor suppressor, decreasing GSH levels in tumor cells, inducing mitochondrial apoptosis and autophagy, and reducing tumor cell proliferation in vitro and in vivo [27,29,30].

**Table 9:** GSH-GSSG system in ccRCC.

GSH-GSSG System Activities	GSH, GSSG and GSH/GSSG Ratio in ccRCC	Our Study	Literature
Carcinogenesis	GSH metabolism is altered in renal tumorigenesis. Its synthesis needs glutamic acid, cysteine and glycine, expression of synthesis enzymes and regeneration of GSH (GCL, GLSL, GS, GGT, GR).	High levels of GSH, GSSG and GSH/GSSG in ccRCC (Table 1) were associated with ROS, RNS, RCS overproduction (Table 2, Table 3 and Table 4) and inflammation, hypoxia and angiogenesis overexpression (Table 5 and Table 6).	GCL protein, GLS1, glutamine importers, cysteine antiporter xCT induce GSH synthesis. Increased PPP flux to produce NADPH for GSH conversion [3,25,26,29,31]. γ-glutamyl isopeptide bond stabilizes GSH in systemic circulation. Transporters expression mediates compensatory increase in GSH in proximal tubular cells; process associated with carcinogenesis [8,25].
Cytoprotective effects	γ-glutamyl cycle could influence renal cells vulnerability to oxidant and electrophile agents.	GSH and GSSH were overexpressed in hypoxic and antioxidative capacity environment (Table 2, Table 3, Table 4, Table 5 and Table 6). GSH/GSSG ratio was positively correlated with oxidation markers (ROS, RNS, RCS) and hypoxia (HIF1a) and negatively with ImAnOx (Table 8).	GSH conjugation with electrophile xenobiotics and inactivation of endogen oxidated species are high in ccRCC, via Nrf2/NF-κB [8,31]. GSH could be considered a protector antioxidant and a bioactivation promotor [8].
Inflammatory and antioxidative response	The redox equilibrium mediated by GSH-GSSG is altered in ccRCC. GSH is a redox cofactor for GPXs, GSTs, GRXs.	IL-12p70, IL-12p40, IL-23 had divergent activities in relation to IL-12p35, IL-35 in ccRCC (Table 5). IL-12p70, IL-12p40, IL-23 had high levels and were associated with GSH increase, while IL-12p35, IL-35 had low levels and were associated with GSSG (Table 8).	In ccRCC, GSH induces an adaptative anti-inflammatory and antioxidant response at high levels of electrophiles and oxidants and limits their toxicity [29]. GSH modulates IL-12 secretion [31] and acts as an inflammation suppressor regulating GSH PTEN/PI3K/AKT pathway [32].
Angiogenesis promotion	GSH mediates interactions between tumor angiogenesis and its hypoxic environment.	HIF1a, HIF2a, VEGF were overexpressed in ccRCC (Table 6). HIF-1a and VEGFs were correlated with GSH and GSSG variations (Table 8).	VHL could repress expression for over 100 genes that interact with HIF-1α and HIF-1AN [31]. GCLM and SLC7A11 expression is regulated by HIF-1α, controlling GSH synthesis in hypoxic conditions [25]. VEGF correlates with GSH, folates and antioxidant enzymes in tumors promoting angiogenesis [31].
Apoptosis limitation	High levels of GSH are an anti-apoptotic signal in ccRCC.	Caspase-3 protein was not correlated with GSH-GSSG levels (Table 8).	GSH-GSSG alters apoptotic process mediated by caspase [3,27,30,33]. GSH protects active redox cysteine in catalytic sites of caspases [3], while GSSG activates caspase-3 [27].
Glutathione restauration—a therapeutic strategy for preventing oxidative/electrophile stress in ccRCC?	γ-glutamyl cycle was involved in electrophile detoxification, modulation if redox-regulated transduction system.	Future studies are needed in order to develop new therapeutic strategies.	Actual antitumoral strategies involve GSH, GCL, GGT, xCT, GLUT, NRF2 [12].

Clear cell renal cell carcinoma (ccRCC); reduced glutathione (GSH); oxidized glutathione (GSSG); antioxidative capacity (ImAn Ox or TAS/TAC); hydroxynonenal (HNE); interleukin (IL); hypoxia inducible factor (HIF); vascular endothelial growth factor (VEGF); statistical significance (*p*); correlation coefficient (r); L-γ-glutamyl-L-cysteinyl-glycine γ (GSH); oxidized glutathione (GSSG); γ -glutamyl-cysteine ligase (γGCL); catalytic subunit (GCLC) and a modulating subunit (GCLM) of γGCL; GSH synthetase (GS); γ glutamyl transferase (GGT); glutathione reductase (GR), glutaminase (GSL); glutathione peroxidases (GPXs); glutathione S-transferases (GSTs) and glutaredoxins (GRXs); pentose phosphate pathway (PPP); transcription factors nuclear erythroid 2-related factor 2 (Nrf2); nuclear factor kappa-light-chain-enhancer of activated B cells (NF-κB).

Oxidative injuries represent high-risk factors for neoplastic development. The γ-glutamyl cycle could influence renal cells’ vulnerability to oxidants and electrophiles in ccRCC. The present study showed high levels of GSH, GSSG and GSH/GSSG ratio in neoplastic subjects, with decreased albumin and ImAnOx. Our findings demonstrated that GSH exerts protective cellular effects in ccRCC. Although albumin has antioxidant properties binding the reactive species and the metal ions, it has no specificity as a marker of oxidative injury [34]. The coordination between the citric acid cycle and oxidative phosphorylation ensured an increase in the adaptive responses of GSH against toxic compounds via Nrf2/NF-κB [8,31]. GSH functioned as a cellular protector and, under certain conditions, as a promoter of bioactivation in other renal pathologies [8]. The distribution of GSH between intracellular compartments played a protective role in cancer initiation and promoted neoplastic progression and resistance acquisition [12]. As a cellular protector, GSH participated in post-translational covalent modifications through S-glutathionylation, which influenced cellular signaling. Additionally, GSH modulated gene expression and protein expression through non-covalent binding to proteins [35,36].

Currently, thiol-dependent antioxidant systems could function as a switch in chronic inflammation by modulating proteins and enzymes that target redox signaling in biology. Pro-inflammatory cytokines, along with reactive species ROS/RNSRCS, RSS, play a crucial role in the adaptive response of the GSH-GSSG system in cancer cells. In the present study, IL-12p70, IL-12p40, and IL-23 displayed an upward profile and were strongly associated with increased GSH, while IL-12p35 and IL-35 functioned as anti-inflammatory agents and were negatively correlated with GSSG levels. This interdependence was ensured by the fact that GSH modulated IL-12 secretion through the activation of p38 MAPK and modulation of IFN-gamma [37]. GSH eliminated inflammation by regulating the production of interleukins, chemokines, NO, TNF alpha, the polarization of M1/M2, and the Th1/Th2 balance via NFKB and Nef2/heme oxygenase 1 and NFKb [25,34,38]. The high GSH/GSSG ratio determined in ccRCC could be explained by GSH’s ability to be oxidized by electrophilic substances in order to exercise antioxidant effects [39]. The regeneration of GSH was associated with PPP activation that produced NADPH(H+) necessary for GSSG conversion to GSH [40]. As an anti-inflammatory, GSH could act as an inflammatory suppressor by downregulating the PTEN/PI3K/AKT pathway [32]. As a primary cellular antioxidant, GSH ensures redox homeostasis, protection of protein thiols, detoxification through conjugation with electrophiles, and reduction in oxidants [12,41].

Our results reconfirmed the proangiogenic effects of GSH in cancer. HIF-1a, HIF-2a, and VEGF were overexpressed in ccRCC and were positively correlated with variations in GSH, GSSG, and the GSH/GSSG ratio. VHL can repress the transcription of over 100 target genes by interacting with HIF-1α and HIF-1AN [3]. The expression of GCLM and SLC7A11 genes was regulated by HIF-1α, thus controlling GSH synthesis in hypoxic environments [25]. VEGF correlated with GSH, folate, and antioxidant enzymes in tumors. VEGF increased hypoxic angiogenesis [31,42,43]. VHL mutations led to HIF-1a and HIF-2a accumulation and created a state of pseudohypoxia, which induced angiogenesis, epithelial–mesenchymal transition, invasion, and metastasis. HIF-1a was documented as a tumor suppressor, while HIF-2a as an oncogene in ccRCC. HIF-1a controlled micro RNA, and HIF-2a regulated the cell cycle and progression of ccRCC [19,21,22]. The intracellular accumulation of HIF-1a and HIF-2a regulated VEGF expression [21].

GSH plays a significant role in apoptosis, necroptosis, ferroptosis, and autophagy [3,44]. In cancer, usually a low GSH/GSSG ratio is expected due to the oxidation of the intracellular redox reserve, while in our study this ratio increased as a metabolic adaptation of tumoral cells through increased biosynthesis of glutathione to support antioxidant defense and maintain redox balance [45].

In the current study, caspase-3 was presented independently of GSH-GSSG fluctuations, as we did not determine the enzymatic activity. Caspase-3 protein could serve as a reservoir for the active form of the functional enzyme. Our data expressed that GSH exerted anti-apoptotic effects in ccRCC. As an anti-apoptotic factor, high levels of GSH had provided protection against stress-induced apoptosis through post-translational regulatory mechanisms [46,47]. Moreover, GSH ensured the maintenance of active redox cysteines at the catalytic sites of caspases [32,33].

Our study showed the anti-inflammatory, antioxidant, anti-apoptotic, and proangiogenic effects of GSH in ccRCC. The glutathione redox system constituted a major element for intracellular homeostasis of GSH, which was maintained through de novo synthesis, regeneration from GSSG, and extracellular absorption of GSH. Understanding the regulation and dynamics of GSH in the cellular environment has clinical importance. Restoring GSH metabolism, subcellular compartmentalization and transport (GCL, GGT, xCT, GLUT, NRF2) could help the anti-tumor strategies for preventing oxidative/electrophilic stress and progression of ccRCC.

Although our study was the first one in the literature that evaluated markers of glutathione redox homeostasis status in ccRCC, this project had some limitations. First of all, it was a monocentric prospective study. Secondly, the monitoring criteria for ccRCC did not include the assessment of the molecules both in blood and urine. Moreover, measuring caspase-3 alone does not allow for definitive conclusions about the regulation of apoptosis, with assessment of other factors involved in the apoptosis cascade being needed. In addition, we did not have data on genetic mutations, and patients were not regularly followed up for a long period of time, in order to analyze the progression/prognosis of ccRCC.

Despite the limitations, the study evaluated a wide range of metabolites, with a representative sample size. The blood samples were collected at baseline from all participants and the biological samples were analyzed in the same laboratory, using the same protocol and at the same time.

In the complicated landscape of ccRCC metabolism, this research offers new insights into GSH metabolism reprogramming in ccRCC pathogenesis. The cells required high levels of GSH to combat electrophilic species. A significantly increased level of GSH identified in this study could be considered a distinctive marker for ccRCC. Thus, the dysregulation of intracellular GSH made tumor cells more susceptible to oxidative/electrophilic stress, angiogenesis, and apoptosis. Consequently, GSH provided a protective effect in ccRCC, but further studies are needed to clarify a potential therapeutic role of GSH in ccRCC.

## 4. Materials and Methods

### 4.1. Study Participants

A prospective, case–control study was developed for a period of three years (September 2022–August 2025) and included 92 subjects diagnosed with primary ccRCC after tumor biopsy and 40 healthy subjects. The study was conducted in accordance with the Declaration of Helsinki and approved by the hospital’s Ethics Committee (1124/22 September 2022). Patients were selected from those who attended the Clinical Hospital of Nephrology “Carol Davila” and Clinical Hospital “Victor Babes”.

A total of 123 subjects with renal tumors were initially identified by ultrasonography and underwent an imagistic exam (computed tomography or nuclear magnetic resonance). From the subjects initially included in the study, 106 were detected with renal tumors under 7 cm, with no secondary determinations. The histological exams of the tumors indicated: 92 clear cell renal cell carcinoma, 9 urothelial tumors, 3 angiolipoma, 1 oncocytoma and 1 papillary carcinoma.

We enrolled healthy subjects and patients over 18 years old with adequate nutritional status. All the subjects signed the informed consent before being enrolled in the study. The exclusion criteria were age < 18 years old, pregnancy, tobacco use, vitamin or antioxidants use, advanced ccRCC, hyperhomocysteinemia or any other cardiovascular, hepatic, thyroid, gastrointestinal, or oncological disease, and any viral or bacterial infections in the previous three months.

The characteristics of the groups are presented in Table 10.

### 4.2. Laboratory Data

**Figure 1:**
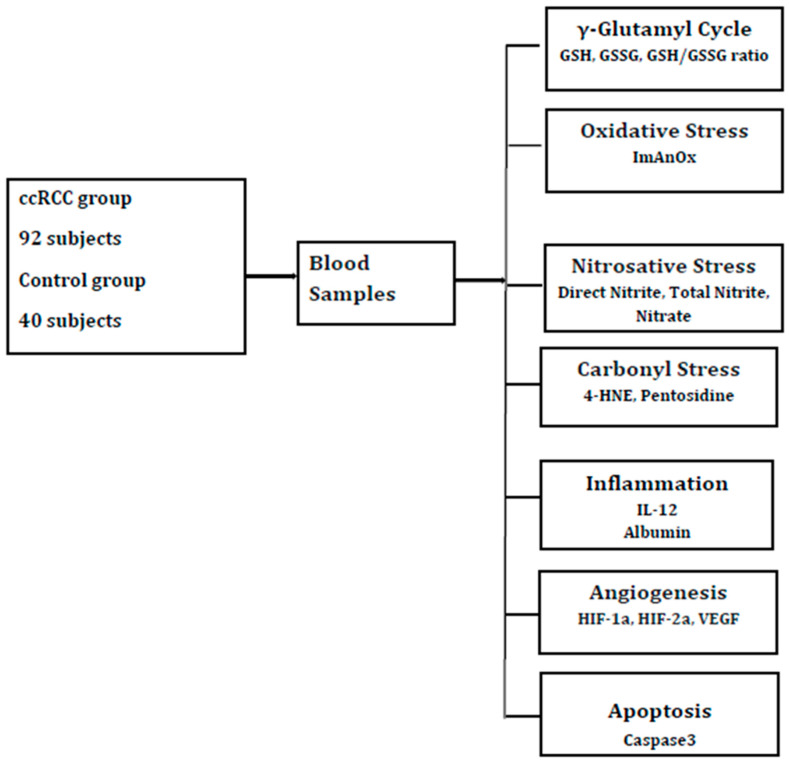
Experimental design. Clear cell renal cell carcinoma (ccRCC); reduced glutathione (GSH); oxidized glutathione (GSSG); antioxidative capacity (ImAnOx or TAS/TAC); hydroxynonenal (HNE); interleukin (IL); hypoxia inducible factor (HIF); vascular endothelial growth factor (VEGF).

The blood samples were collected in the 92 ccRCC subjects before surgical removal of the tumor and in 40 healthy participants. All the procedures were done after the subjects signed the informed consent. Before blood collection, all the study participants underwent 12 h of fasting. The blood samples were centrifugated for ten minutes at room temperature and frozen for one hour. The hemolyzed, icteric, lactescent, or microbiologically contaminated samples were excluded. After collecting the samples, they were sent to evaluate the markers for glutathione redox homeostasis, oxidative stress, nitrosative stress, carbonyl stress, inflammation and angiogenesis as presented in Figure 1.

The main laboratory determinations were markers of glutathione redox homeostasis activity (GSH, GSSG) by colorimetric assay (E-BC-KO97-M kit, Elabscience, Wuhan, Hubei, China); antioxidative capacity (ImAnOx by colorimetric technique using Immundiagnostik KC5200 kit (Bensheim, Germany); albumin by spectrophotometric method using commercial kits with green bromocresol, evaluated at 660 nm), nitrosative stress (direct nitrite, total nitrite, nitrate) assessed by colorimetric method using Cayman Chemical CAY780001 kit, Ann Arbor, MI, USA). Carbonyl stress was assessed by ELISA method (4-HNE—Elabscience Elisa E-EL-0128 kit, Pentosidine—Elabscience Elisa E-EL-0091 kit, Wuhan, Hubei, China). The angiogenesis factors were determined using ELISA method, as follows: HIF-1a by MBS2885065 kit (San Diego, CA, USA), HIF-2a by MBS2022322 kit (San Diego, CA, USA) and VEGF using RDSystems DVE00 Quantikinekit (Minneapolis, USA, Minnesota). The apoptosis-related markers were assessed by ELISA method; caspase was assessed by Human Caspase-3 (Hu Casp 3) FineTest EH0546 kit (Wuhan, China).

IL-12 monomers and heterodimers were assessed by ELISA method, using the following ELABSCIENCE kits (Wuhan, Hubei, China): HumanIL-12p70 ELISA kit, Cat.No.:E-EL-H0150; HumanIL-12p35 ELISA kit, Cat.No.:E-EL-H1647, HumanIL-12p40 ELISA kit Cat.No.:E-EL-H0151, HumanIL-23 ELISA kit Cat.No.:E-EL-H0107 and HumanIL-35 ELISA kit Cat.No.:E-EL-H2443. IL-12 cytokine family has been studied as an inflammatory marker by the authors in other pathologies (autosomal dominant polycystic kidney disease, chronic urticaria) in relation with factors of inflammation (C reactive protein, IL-6, soluble IL-6R, IL-8, fibrinogen), acting as a messenger that initiates, amplifies, and orchestrates the body’s immune-inflammatory response in the presence of a trigger [48,49].

### 4.3. Statistical Analysis

Mean and standard deviation were used for presenting the data. *p* = 0.05 was chosen as level of signification and the confidence interval was 95% for hypothesis testing. The data analysis between groups was based on *t*-tests. After assessing the data normality by the Kolmogorov–Smirnov test, Spearman’s correlation coefficient was used for evaluating the relations between the studied molecules

## 5. Conclusions

The dynamic interaction between bio reactive species and redox signaling mechanisms became an essential research area in carcinogenesis. A high level of oxidative/electrophile stress (ROS, RNS, RCS, RSS) documented by the present study in ccRCC was a fundamental characteristic for maintaining viability and increase in malignant cells. Our study showed significant variations in reactive biological species (GSH, GSSG, ImAnOx, nitrites, nitrates, 4-HNE, pentosidine), hypoxia-associated molecular factors (HIF1a, HIF2a), inflammation (IL-12p70, IL-12p40, IL-23 IL-12p35, IL-35, albumin), apoptosis (caspase-3), and angiogenesis (VEGF) in ccRCC.

GSH-GSSG redox activity was altered in localized ccRCC by metabolic reprogramming and high turnover of GSH, high levels of GSH/GSSG, and decreased consumption of GSH; GSH exerts protective, anti-inflammatory, antioxidant, anti-apoptosis and angiogenic effects. These findings are consistent with increased oxidative stress in tumor tissue, potentially accompanied by a compensatory upregulation of GSH synthesis, involving activation of the NRF2 pathway.

The results of the present study bring a new perspective in ccRCC pathogenesis, therapeutic approaches and diagnosis. More prospective multicenter studies are needed for validating the hypothesis that glutathione restauration could be a therapeutic approach in preventing oxidative/electrophile stress in ccRCC.

## Figures and Tables

**Table 1:** Laboratory reactive sulfur species metabolites in ccRCC and control group.

Biological Parameters	ccRCC Group (*N* = 92)	Control Group (*N* = 40)	*p* Values
GSH (µmol/L)	29.9 ± 7.3	18.0 ± 2.4	0.001
GSSG (µmol/L)	9.1 ± 3.4	6.6 ± 0.8	0.005
GSH/GSSG	3.3 ± 0.4	2.7 ± 0.2	0.002

Reactive sulfur species (RSS); clear cell renal cell carcinoma (ccRCC); reduced glutathione (GSH); oxidized glutathione (GSSG); statistical significance (*p*).

**Table 2:** Laboratory reactive oxygen species indicators in ccRCC and control group.

Biological Parameters	ccRCC Group (*N* = 92)	Control Group (*N* = 40)	*p* Values
ImAnOx (umols/L)	201.5 ± 47.1	299.4 ± 14.7	0.002

Reactive oxygen species (ROS); clear cell renal cell carcinoma (ccRCC); antioxidative capacity (ImAn Ox or TAS/TAC); statistical significance (*p*).

**Table 3:** Laboratory reactive nitrogen species metabolites in ccRCC and control group.

Biological Parameters	ccRCC Group (*N* = 92)	Control Group (*N* = 40)	*p* Values
Direct nitrite (umols/L)	38.2 ± 8.4	15.9 ± 2.6	0.008
Total nitrite (umols/L)	87.3 ± 14.9	33.7 ± 5.6	0.001
Nitrate (umols/L)	49.1 ± 5.8	17.8 ± 4.2	0.021

Reactive nitrogen species (RSS); clear cell renal cell carcinoma (ccRCC); statistical significance (*p*).

**Table 4:** Laboratory reactive carbonyl species indicators in ccRCC and control group.

Biological Parameters	ccRCC Group (*N* = 92)	Control Group (*N* = 40)	*p* Values
4-HNE (ug/mL)	34.1 ± 8.2	14.7 ± 3.8	0.002
Pentosidine (pg/mL)	1706.2 ± 916.8	1108.9 ± 432.6	0.001

Reactive carbonyl species (RCS); clear cell renal cell carcinoma (ccRCC); hydroxynonenal (HNE); statistical significance (*p*).

**Table 5:** Laboratory inflammatory-related proteins in ccRCC and control group.

Biological Parameters	ccRCC Group (*N* = 92)	Control Group (*N* = 40)	*p* Values
IL-12p70 (pg/mL)	36.3 ± 15.6	17.7 ± 4.08	0.001
IL-12p35 (pg/mL)	15.6. ± 5.9	22.7 ± 3.1	0.02
IL-12p40 (pg/mL)	225.3 ± 77.3	74.8 ± 15.7	0.001
IL-23 (pg/mL)	73.7 ± 24.8	30.7 ± 4.8	0.012
IL-35 (pg/mL)	9.2 ± 4.4	11.6 ± 0.4	0.053
Albumin (g/dL)	3.7 ± 0.7	4.1 ± 0.4	0.040

Clear cell renal cell carcinoma (ccRCC); interleukin (IL); statistical significance (*p*).

**Table 6:** Laboratory angiogenesis-related factors in ccRCC and control group.

Biological Parameters	ccRCC Group (*N* = 92)	Control Group (*N* = 40)	*p* Values
HIF-1a (ng/mL)	99.8 ± 21.1	53.4 ± 6.9	0.008
HIF-2a (ng/mL)	2.2 ± 0.7	1.4 ± 0.5	0.041
HIF-1a/HIF-2a	45.4 ± 4,4	39.5 ± 4.8	0.006
VEGF (pg/mL)	443.3 ± 89.7	128.6 ± 27.9	0.003

Clear cell renal cell carcinoma (ccRCC); hypoxia inducible factor (HIF); vascular endothelial growth factor (VEGF); statistical significance (*p*).

**Table 7:** Laboratory apoptosis-related marker in ccRCC and control group.

Biological Parameters	ccRCC Group (*N* = 92)	Control Group (*N* = 40)	*p* Values
Caspase-3 (pg/mL)	2.4 ± 1.0	1.3 ± 0.4	0.048

**Table 8:** Statistical relations between markers of glutathione redox homeostasis and the studied parameters in ccRCC.

Parameters	GSH		GSSG		GSH/GSSG	
	r	*p*	r	*p*	r	*p*
ImAnOx	−0.615	0.001	−0.287	0.034	−0.722	0.003
Albumin	−0.261	0.018	−0.102	0.131	−0.199	0.071
Direct nitrite	0.253	0.052	0.161	0.074	0.128	0.372
Total nitrite	0.353	0.034	0.275	0.088	0.419	0.003
Nitrate	0.163	0.026	0.452	0.024	0.147	0.250
4-HNE	0.543	0.004	0.632	0.02	0.679	0.003
Pentosidine	0.825	0.001	0.205	0.045	0.774	0.001
IL-12p70	0.525	0.002	0.177	0.321	0.295	0.211
IL-12p35	−0.131	0.677	−0.126	0.004	−0.102	0.243
IL-12p40	0.306	0.077	0.111	0.532	0.173	0.302
IL-23	0.128	0.033	0.144	0.07	0.143	0.231
IL-35	−0.222	0.061	−0.409	0.045	−0.082	0.721
HIF-1a	0.528	0.012	0.375	0.024	0.195	0.033
HIF-2a	0.341	0.026	0.205	0.032	0.445	0.015
VEGF	0.536	0.027	0.611	0.031	0/173	0.546
Caspase2	0.048	0.864	0.044	0.454	−0.143	0.286

Clear cell renal cell carcinoma (ccRCC); reduced glutathione (GSH); oxidized glutathione (GSSG); antioxidative capacity (ImAn Ox or TAS/TAC); hydroxynonenal (HNE); interleukin (IL); hypoxia inducible factor (HIF); vascular endothelial growth factor (VEGF); statistical significance (*p*); correlation coefficient (r).

**Table 10:** Patients’ characteristics in the studied groups.

Characteristics	ccRCC (*N* = 92)	Control (*N* = 40)	*p* Value
Women:Men	43/49	19/21	0.26
Age (years old)	54.2 ± 9.7	46.8 ± 11.3	0.05
BMI (Kg/mp)	23.4 ± 1.7	22.7 ± 2.4	0.19
Systolic Pressure (mmHg)	12.1 ± 1.4	11.4 ± 1.3	0.34
Diastolic Pressure (mmHg)	5.9 ± 1.1	6.1 ± 0.6	0.28
Leucocytes (cells/mmc)	6800 ± 2104	6230 ± 1130	0.45
Erythrocytes (10^3^ cells/mmc)	4870 ± 392	5100 ± 904	0.57
Calcium (mg/dL)	9.43 ± 0.6	9.04 ± 0.8	0.12
LDH(U/L)	307 ± 71	292 ± 54	0.63
Glycemia (mg/dL)	84.7 ± 11.2	80.3 ± 13.2	0.27
eGFR (ml/min/1,73 mp)	90.4 ± 12.7	96.22 ± 20.12	0.15
Uric Acid (mg/dL)	4.7 ± 2.4	4.1 ± 1.6	0.39
ASAT (U/L)	14.6 ± 12.2	16.7 ± 10.5	0.71
ALAT (U/L)	20.1 ± 14.1	26.2 ± 6.2	0.43
Cholesterol (mg/dL)	137.3 ± 32.4	142.6 ± 27.2	0.18
Triglycerides (mg/dL)	77.5 ± 22.4	86.3 ± 13.6	0.36
Albumin (g/dL)	3.78 ± 0.29	3.91 ± 0.37	0.04
UACR (mg/g creatinine)	9.7 ± 4.25	8.12 ± 3.77	0.07
CRP (mg/dL)	2.66 ± 0.59	0.44 ± 0.23	0.01

ccRCC—clear cell renal cell carcinoma, *p*—statistical significance, BMI—body mass index, LDH—lactate dehydrogenase, eGFR—estimated glomerular filtration rate, ASAT—aspartate aminotransferase, ALAT—alanine aminotransferase, UACR—urinary albumin creatinine ratio, CRP—C reactive protein.

## Data Availability

The original contributions presented in this study are included in the article. Further inquiries can be directed to the corresponding authors.
